# Blinatumomab in pediatric patients with relapsed/refractory acute lymphoblastic leukemia: results of the RIALTO trial, an expanded access study

**DOI:** 10.1038/s41408-020-00342-x

**Published:** 2020-07-24

**Authors:** Franco Locatelli, Gerhard Zugmaier, Noemi Mergen, Peter Bader, Sima Jeha, Paul-Gerhardt Schlegel, Jean-Pierre Bourquin, Rupert Handgretinger, Benoit Brethon, Claudia Rossig, Christiane Chen-Santel

**Affiliations:** 1 Department of Hematology and Oncology, IRCCS Bambino Gesù Children’s Hospital, Rome, Sapienza, University of Rome, Rome, Italy; 2 Amgen Research (Munich) GmbH, Munich, Germany; 3 Department for Children and Adolescents, University Hospital Frankfurt, Frankfurt, Germany; 4 St Jude Children’s Research Hospital, Memphis, TN USA; 5 University Children’s Hospital Wuerzburg, Wuerzburg, Germany; 6 Department of Pediatric Oncology, Children’s Research Centre, University Children’s Hospital Zurich, Zurich, Switzerland; 7 Department of Hematology/Oncology, University Children’s Hospital Tuebingen, Tuebingen, Germany; 8 Pediatric Hematology and Immunology Department, Robert Debre Hospital, APHP, Paris, France; 9 Department of Pediatric Hematology and Oncology, University Children’s Hospital Muenster, Muenster, Germany; 10 Department of Pediatrics, Division of Oncology and Hematology, Charité Universitätsmedizin Berlin, Berlin, Germany

**Keywords:** Cancer therapy, Cancer

Dear Editor,

Although most children with B-cell precursor acute lymphoblastic leukemia (BCP-ALL) achieve first remission with conventional, risk-adapted protocols, relapse still occurs in 15–20% of patients^1^. Current salvage treatments are associated with acute and long-term toxicities, which may cause long-term sequelae and treatment-related (TR) death^2^. As minimal residual disease (MRD) is a strong predictor of relapse in BCP-ALL^3^, new treatments associated with reduced toxicity and high rates of MRD clearance are needed to improve outcomes for children with relapsed/refractory (r/r) BCP-ALL.

Blinatumomab is a bispecific CD19-directed CD3 T-cell engager (BiTE) molecule shown to improve overall survival (OS) as compared with standard chemotherapy in adult patients with r/r BCP-ALL, and to induce high rates of complete remission (CR) and complete MRD response^4–6^. An international phase 1/2 study established the recommended dose of blinatumomab in children and adolescents with r/r BCP-ALL, and demonstrated its antileukemic activity across age and risk groups^7^.

We report on the safety and efficacy of blinatumomab in an open-label, single-arm, expanded access international study of pediatric patients with CD19-positive r/r BCP-ALL (RIALTO trial, NCT02187354). The protocol was approved by the Institutional Review Board/Independent Ethics Committee at each site; informed consent was obtained from patients’ legal guardians. Enrolled patients were aged >28 days to <18 years, with CD19-positive BCP-ALL in second or later relapse, any relapse after allogeneic hematopoietic stem cell transplantation (alloHSCT), or refractory to other treatments. All patients had ≥5% blasts or <5% blasts but with MRD level ≥ 10^−3^, and adequate liver function at screening. Blinatumomab (5–15 µg/m^2^ per day) was administered as a 6-week induction cycle, comprising continuous infusion for 4 weeks, followed by a 2-week treatment-free period. Up to two induction cycles could be performed. Those achieving CR could then receive up to three additional consolidation cycles for a total of five cycles. Additional eligibility criteria, dosing information, and dose interruption or discontinuation criteria are included in Supplementary Material.

The primary endpoint was incidence of treatment-emergent (TE) and TR adverse events (AEs). Secondary endpoints included morphologic CR (<5% blasts) and MRD response (<10^−4^ leukemic blasts by flow cytometry) in the first two cycles, relapse-free survival (RFS), OS, alloHSCT rate after blinatumomab treatment, and 100-day mortality after alloHSCT. Bone marrow aspirate or biopsy was performed at screening, after blinatumomab infusion on day 29 of each treatment cycle, and 6-monthly during follow-up, until 18 months after the first blinatumomab dose. Statistical analyses are described in Supplementary Material.

The study began in 2014. Data cut-off for the current analysis was July 19, 2019. Baseline demographics and clinical characteristics for all patients (*n* = 110; full analysis set) are shown in Supplementary Table 1. Eleven percent (*n* = 12) had <5% blasts (with MRD ≥ 10^−3^) at baseline. Thirty-two patients remained on treatment at the cut-off date (Supplementary Fig. 1).

Almost all patients (99%) experienced TEAEs (Supplementary Table 2); AEs of ≥grade 3 were experienced by 65% of patients. TRAEs were reported in 74% of patients; 26% were ≥grade 3 (Supplementary Table 2). TRAEs leading to blinatumomab discontinuation or temporary interruption occurred in 4% and 16% of patients, respectively. There were nine fatal AEs; none were attributed to blinatumomab. Forty-two percent of patients experienced neurologic AEs, most commonly headache (25%). Six patients (5.5%) experienced grade 3 neurologic events: two patients experienced headache, and one patient each had depressed level of consciousness, seizure, trigeminal nerve disorder, and agitation. AEs were managed per protocol (see “Treatments” in Supplementary Material). There were no grade 4 or 5 neurologic AEs. Two patients (1.8%) experienced grade ≥ 3 cytokine release syndrome (CRS), and one (1%) had grade 4 CRS.

Among patients with ≥5% blasts at baseline (*n* = 98), 58 patients (59%) achieved CR within the first two blinatumomab cycles. Of these, 39 (67%) also achieved full hematologic recovery and 46 (79%) achieved MRD response (Supplementary Table 3). Details of patients with nonevaluable or unavailable response data are given in the Supplementary Material. Of the twelve patients enrolled with <5% blasts, 11 (92%) achieved MRD response during the first two blinatumomab cycles including three with full recovery of peripheral blood counts, while response data were unavailable for one patient.

Overall, the response rate was higher among patients with lower baseline tumor burden (Fig. 1). Four of five patients with prior blinatumomab exposure achieved CR with full recovery of peripheral blood counts, and three achieved MRD response in the first two cycles. Two patients had t(17;19) rearrangement and both achieved CR with MRD response in the first two treatment cycles; both completed the study in remission and were alive at last follow-up. Three of the four patients with constitutional trisomy 21 achieved CR with MRD response, two completed the study in remission and were still alive at last follow-up.

**Fig. 1:**
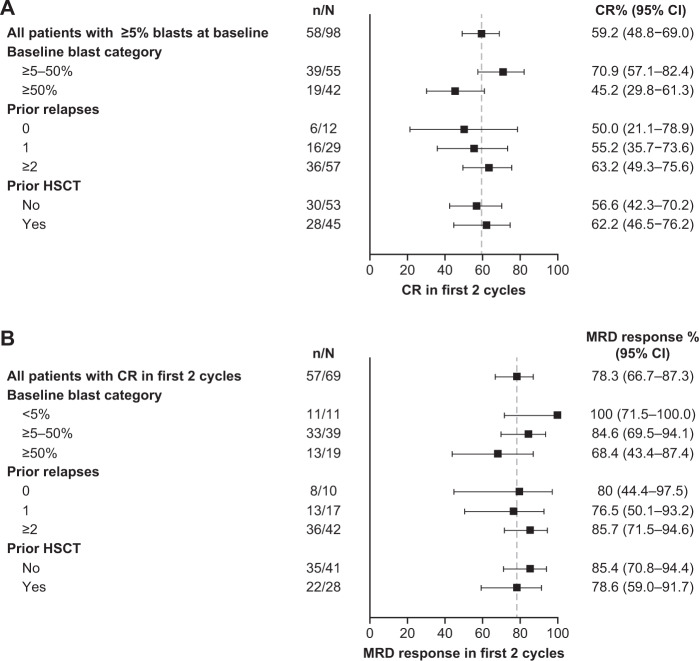
Response rates within the first 2 cycles of blinatumomab treatment, by subgroup. **a** Complete remission (CR) rates in patients with ≥5% blasts at baseline. **b** Minimal residual disease (MRD) response rates.

Of 110 patients in the study, 69 patients had CR as best response in the first two cycles; of these, 45 (65%) proceeded to alloHSCT. Simon–Makuch 42-day landmark analyses showed a trend toward improved OS and RFS for patients who received alloHSCT after blinatumomab as compared with those who did not (Fig. 2e, f). The Kaplan–Meier estimate of 100-day mortality following alloHSCT for these patients (*n* = 45) was 4.5% (95% CI 1.2–17.0%). Six of twenty-three relapses (26%) were CD19-negative.

**Fig. 2:**
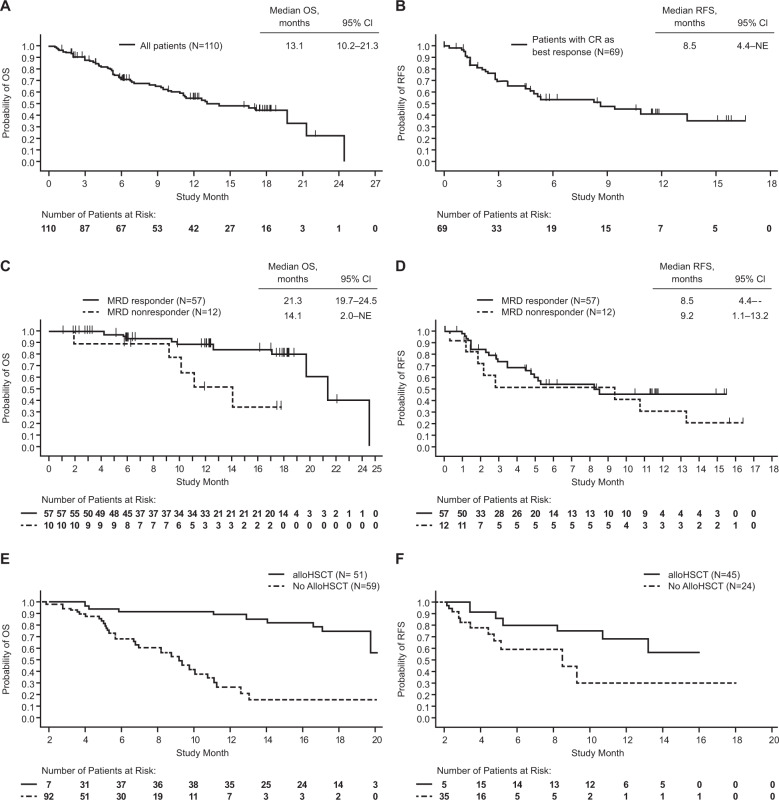
Kaplan–Meier and Simon–Makuch analyses of overall survival (OS) and relapse-free survival (RFS). For the Kaplan–Meier analyses, OS was analyzed in the full analysis set, overall (**a**) and according to the minimal residual disease (MRD) response (**c**), and RFS was analyzed in all patients who achieved complete remission (CR), overall (**b**) and according to the MRD response (**d**), calculated from time of CR. For the Simon–Makuch analyses, both OS (**e**) and RFS (**f**) were analyzed according to the alloHSCT status post blinatumomab. RFS is calculated only on patients with CR.

Median OS for all patients (*n* = 110) was 13.1 months (95% CI 10.2–21.3), with a median follow-up of 17.4 months (Fig. 2a). For all patients reaching or maintaining CR in the first two cycles of blinatumomab (*n* = 69), median RFS was 8.5 months (95% CI 4.4—not evaluable), with a median follow-up of 11.2 months (Fig. 2b); 23 patients (33%) relapsed and 6 (9%) died. Those who achieved MRD response had longer OS than those who achieved CR without MRD response; however, there was no apparent difference in RFS between these subgroups (Fig. 2c, d). For patients given HSCT in continuous CR, OS was nonestimable at the 15-month analysis for both the MRD responders and nonresponders groups (Supplementary Fig. 2).

Our findings demonstrate an acceptable safety profile and high response rate with blinatumomab in pediatric patients with r/r BCP-ALL. Blinatumomab is generally better tolerated compared with chemotherapy^5,8,9^; however, it is associated with distinct AEs, including neurologic toxicities and CRS. In this study, such events at ≥grade 3 were infrequent. Incidence of grade 3/4 AEs was lower than in the recent phase 2 pediatric study^7^, possibly reflecting the lower tumor burden of patients recruited here. Overall, the safety profile was consistent with that reported in other studies of blinatumomab^4,6,7^.

Our efficacy findings showed a trend similar to that observed in the recent phase 2 study of blinatumomab in pediatric patients with r/r BCP-ALL, in which a higher CR rate was seen in patients with lower baseline blast counts^7^. This explains the increased response rate observed here compared with the phase 2 trial, since a higher proportion of patients with <50% blasts were enrolled (61% of patients versus 26% in the phase 2 study)^7^.

Blinatumomab demonstrated a 78% molecular response rate in adult patients in morphological remission (<5% blasts with MRD levels ≥10^−3^);^4^ ours is the first pediatric study to include patients with these disease characteristics. Of the 12 patients recruited, 11 achieved MRD response within the first two blinatumomab cycles, suggesting that further investigation in this patient population is warranted.

There was no apparent difference in response rates according to the number of prior relapses or prior alloHSCT. This contrasts with chemotherapy outcomes, where response rates decline with each consecutive relapse^10^. Only five patients had received prior blinatumomab, but four of five achieved CR, and three achieved MRD response, indicating that reexposure to blinatumomab may be effective, provided that leukemia blasts are still CD19-positive; CD19-negative relapse, a tumor-escape mechanism, indicates a poor prognosis^11^. Achievement of CR in three of four patients with constitutional trisomy 21 suggests that blinatumomab could be particularly useful to treat these fragile patients at risk of experiencing severe toxicities when exposed to aggressive chemotherapy^12^. Remarkably, both patients with t(17;19) rearrangement obtained CR with MRD response, this finding corroborating recent data on blinatumomab efficacy in this peculiar patient subset^13^.

A recent meta-analysis reported a significant OS benefit for pediatric patients who achieved MRD negativity relative to those who did not^3^. Here, Kaplan–Meier analyses show improved OS for patients who achieved MRD response as compared with those who did not, with no difference in RFS, possibly because of the low number of patients in the MRD nonresponder group (*n* = 12). Among patients with CR as best response in the first two blinatumomab cycles, 65% proceeded to alloHSCT, and these patients appeared to have longer RFS and OS compared with patients who did not subsequently receive alloHSCT, a finding consistent with the phase 2 pediatric trial^7^. Our study is limited by the lack of a comparator arm and the assessment of outcomes by investigators rather than by independent central review.

Targeted immunotherapies other than blinatumomab are being developed and have demonstrated activity in pediatric patients with r/r BCP-ALL. These include inotuzumab ozogamicin, although not approved for pediatric patients^14^, and CD19-directed chimeric antigen receptor T-cell therapies^15–17^. These trials highlight expansion of treatment options beyond standard of care chemotherapeutic approaches in patients with r/r BCP-ALL.

This expanded access study with blinatumomab in pediatric patients with r/r BCP-ALL confirms a tolerable safety profile and indicates a response rate higher than previously reported, further supporting the use of blinatumomab for treatment of children with BCP-ALL.

## Supplementary information

Supplementary Material

## Data Availability

Qualified researchers may request data from Amgen clinical studies. Complete details are available at the following: http://www.amgen.com/datasharing.
