# Author Correction: Blinatumomab in pediatric patients with relapsed/refractory acute lymphoblastic leukemia: results of the RIALTO trial, an expanded access study

**DOI:** 10.1038/s41408-021-00413-7

**Published:** 2021-02-01

**Authors:** Franco Locatelli, Gerhard Zugmaier, Noemi Mergen, Peter Bader, Sima Jeha, Paul-Gerhardt Schlegel, Jean-Pierre Bourquin, Rupert Handgretinger, Benoit Brethon, Claudia Rossig, Christiane Chen-Santel

**Affiliations:** 1 Department of Hematology and Oncology, IRCCS Bambino Gesù Children’s Hospital, Rome, Sapienza, University of Rome, Rome, Italy; 2 Amgen Research (Munich) GmbH, Munich, Germany; 3 Department for Children and Adolescents, University Hospital Frankfurt, Frankfurt, Germany; 4 St Jude Children’s Research Hospital, Memphis, TN USA; 5 University Children’s Hospital Wuerzburg, Wuerzburg, Germany; 6 Department of Pediatric Oncology, Children’s Research Centre, University Children’s Hospital Zurich, Zurich, Switzerland; 7 Department of Hematology/Oncology, University Children’s Hospital Tuebingen, Tuebingen, Germany; 8 Pediatric Hematology and Immunology Department, Robert Debre Hospital, APHP, Paris, France; 9 Department of Pediatric Hematology and Oncology, University Children’s Hospital Muenster, Muenster, Germany; 10 Department of Pediatrics, Division of Oncology and Hematology, Charité Universitätsmedizin Berlin, Berlin, Germany

Correction to: *Blood Cancer Journal*

10.1038/s41408-020-00342-x published online 24 July 2020

The original version of the article contained an error in Fig. 1b, which showed that 57/69 (78.3%) of patients with CR in the first 2 cycles achieved MRD response. The article has now been corrected to state 57/69 (82.6%).

**Figure 1:**
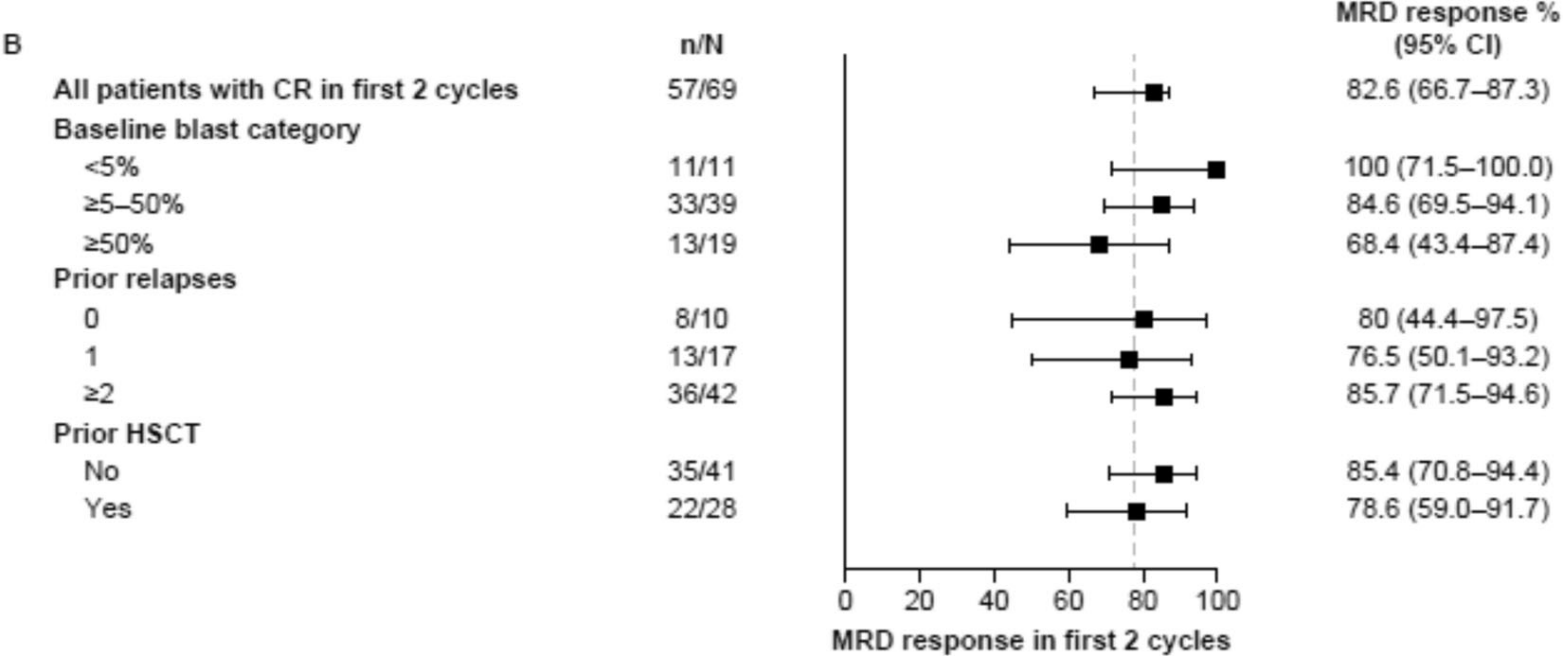
Fig. 1

