# Correction: Blinatumomab in pediatric patients with relapsed/refractory acute lymphoblastic leukemia: results of the RIALTO trial, an expanded access study

**DOI:** 10.1038/s41408-021-00567-4

**Published:** 2021-10-27

**Authors:** Franco Locatelli, Gerhard Zugmaier, Noemi Mergen, Peter Bader, Sima Jeha, Paul-Gerhardt Schlegel, Jean-Pierre Bourquin, Rupert Handgretinger, Benoit Brethon, Claudia Rossig, Christiane Chen-Santel

**Affiliations:** 1 Department of Hematology and Oncology, IRCCS Bambino Gesù Children’s Hospital, Rome, Sapienza, University of Rome, Rome, Italy; 2 Amgen Research (Munich) GmbH, Munich, Germany; 3 Department for Children and Adolescents, University Hospital Frankfurt, Frankfurt, Germany; 4 St Jude Children’s Research Hospital, Memphis, TN USA; 5 University Children’s Hospital Wuerzburg, Wuerzburg, Germany; 6 Department of Pediatric Oncology, Children’s Research Centre, University Children’s Hospital Zurich, Zurich, Switzerland; 7 Department of Hematology/Oncology, University Children’s Hospital Tuebingen, Tuebingen, Germany; 8 Pediatric Hematology and Immunology Department, Robert Debre Hospital, APHP, Paris, France; 9 Department of Pediatric Hematology and Oncology, University Children’s Hospital Muenster, Muenster, Germany; 10 Department of Pediatrics, Division of Oncology and Hematology, Charité Universitätsmedizin Berlin, Berlin, Germany

Correction to: *Blood Cancer Journal* 10.1038/s41408-020-00342-x published online 24 July 2020

Following the publication of this article, the authors requested the following corrections:

Page 2: The sentence “Three of the four patients with constitutional trisomy 21 achieved CR with MRD response, two completed study in remission and were still alive at last follow-up”. changed to “All four patients with constitutional trisomy 21 achieved CR with MRD response, two completed study in remission and were still alive at last follow-up”.

Page 4: The sentence “Achievement of CR in three of four patients with constitutional trisomy 21 suggests that blinatumomab could be particularly useful to treat these fragile patients at risk of experiencing severe toxicities when exposed to aggressive chemotherapy.” changed to “Achievement of CR in all four patients with constitutional trisomy 21 suggests that blinatumomab could be particularly useful to treat these fragile patients at risk of experiencing severe toxicities when exposed to aggressive chemotherapy”.

The authors confirm no change to the conclusions of the article.

