# Supplementary material for: Blinatumomab in pediatric patients with relapsed/refractory acute lymphoblastic leukemia: results of the RIALTO trial, an expanded access study
Source: Blood Cancer J. 2020 Jul 24;10(7):77. doi: 10.1038/s41408-020-00342-x (PMC7381625; doi:10.1038/s41408-020-00342-x)
Supplement: Supplementary file 1 — Supplementary Material [file 41408_2020_342_MOESM1_ESM.pdf]

This file includes additional methods and safety and efficacy results, along with a patient disposition figure, tables displaying baseline demographics and clinical characteristics, adverse events, and responses to blinatumomab treatment, overall and by subgroup.

Supplementary Material

Supplementary Methods

Inclusion criteria

Patients previously treated with blinatumomab were eligible for inclusion provided that they were not intolerant or refractory to blinatumomab, and leukemic cells were still CD19 positive.

Exclusion criteria

Patients with active acute (grade 2–4) or chronic graft-versus-host disease (GvHD) requiring systemic treatment, or active central nervous system or testicular involvement, were excluded. Other key exclusion criteria were receipt of chemotherapy within 2 weeks, radiotherapy within 4 weeks, and immunotherapy within 6 weeks, or systemic immunosuppressive drugs to treat GvHD within 2 weeks of blinatumomab treatment.

Treatments

During a 2-week screening and pre-phase period, patients could receive dexamethasone or hydroxyurea to reduce tumor burden and the risk of tumor lysis syndrome. Dexamethasone was recommended prior to the first dose of blinatumomab and was required for patients with bone marrow blasts >50% at screening. During screening, patients received dexamethasone up to 24 mg/day for up to 4 days. Prophylactic dexamethasone was administered at 10 mg/m^2 from 6 to 12 hours before each infusion, and 5 mg/m^2 within 30 minutes of each infusion.

During induction and consolidation cycles, patients with ≤25% blasts at baseline received blinatumomab 15 µg/m^2 per day; those with >25% blasts at baseline received 5 µg/m^2 on days 1−7 of cycle 1, and 15 µg/m^2 per day thereafter.

Central nervous system prophylaxis was administered according to institutional/national standards within 1 week prior to starting blinatumomab, following each treatment cycle, and after bone marrow aspiration on day 29.

Dosing was interrupted if patients experienced a blinatumomab-related grade 2 clinically relevant neurologic event, grade 2 or higher cytokine release syndrome (CRS), tumor lysis syndrome, or disseminated intravascular coagulation or coagulopathy, or any clinically relevant event of grade 3 or higher. Following resolution to a minimum of grade 1, blinatumomab was restarted at 5 µg/m^2 per day for at least 7 days before increasing to 15 µg/m^2 per day. Blinatumomab was permanently discontinued if a patient experienced an adverse event (AE)-related infusion interruption that lasted

more than 2 weeks. Other criteria for discontinuation included disease progression, extramedullary relapse, or intolerable toxicity.

Assessments

AEs were graded according to National Cancer Institute's Common Terminology Criteria for Adverse Events version 4.03.

Statistical analysis

The primary endpoint of the study was the incidence of treatment-emergent and treatment-related AEs. The primary endpoint focused on safety rather than efficacy because, although blinatumomab has been evaluated previously in a phase 2 study of a pediatric population (von Stackelberg et al, 2016), the children included had different characteristics as compared to those in the present study.

A formal hypothesis was not tested; therefore, statistical reporting of this study is descriptive. The current analysis includes the first 110 patients enrolled. Safety analyses were performed on the full analysis set, which included all patients who received any infusion of blinatumomab. For response endpoints, patients with missing response data were considered non-responders. Survival endpoints were estimated by the Kaplan-Meier method or the Simon-Makuch method, with a 42-day landmark.

Supplementary Results

Safety and exposure

The median number of treatment cycles completed was 1 (range, 1–5). Reasons for discontinuation of blinatumomab included proceeding to allogeneic hematopoietic stem cell transplantation (alloHSCT) (31%), disease progression (24%), hematological or extramedullary relapse subsequent to CR (9%), >25% leukemic blasts at the end of cycle 1 (8%), AEs (6%), requirement for alternative therapy (5%), study completion (5%), failure to achieve CR in the first 2 cycles (3%), and patient request (2%).

Grade 3 nervous system disorders were reported in 5 patients (5%), including 2 patients with headache, and one patient each who had depressed level of consciousness, seizure and a trigeminal nerve disorder, respectively (unrelated to study treatment).

Efficacy

Response data were not evaluable for one patient and unavailable for 2 patients; these were counted as non-responders in analyses. Of the 2 patients without response data, one died due to multiorgan failure and one experienced a leukemia phenotype switch to acute myeloid leukemia (AML). This latter patient was 17-month-old and had mixed lineage leukemia (MLL)-rearranged ALL. This case has been previously described (1).

Among patients with ≥5% blasts at baseline (n=98), 58 (59.2% [95% CI, 48.8%−69.0%]) achieved complete remission during the first cycle of treatment with blinatumomab. Of these, 32 (32.7% [23.5−42.9]) achieved full hematologic recovery and 43 (74.1% [61.0−84.7]) achieved an MRD response.

Six patients who did not achieve CR in the first 2 cycles proceeded to alloHSCT, following additional chemotherapy. Of these, two completed the study, 2 died from disease progression, and 2 died from pulmonary complications. Among patients achieving CR (n=69), 40 (58%) completed the study in remission, 23 (33%) relapsed during follow-up and 6 (9%) died. Of the latter, one died from disease progression, 2 died from infection, 2 died from respiratory distress syndrome and one died from hypovolemic shock. Six of 23 relapses (26%) were CD19 negative, including one AML phenotype switch; this patient discontinued blinatumomab and subsequently had a CD19-positive ALL relapse.(1) CD19 status at the time of relapse was unavailable for one patient.

Supplementary Fig. 1. Patient disposition.

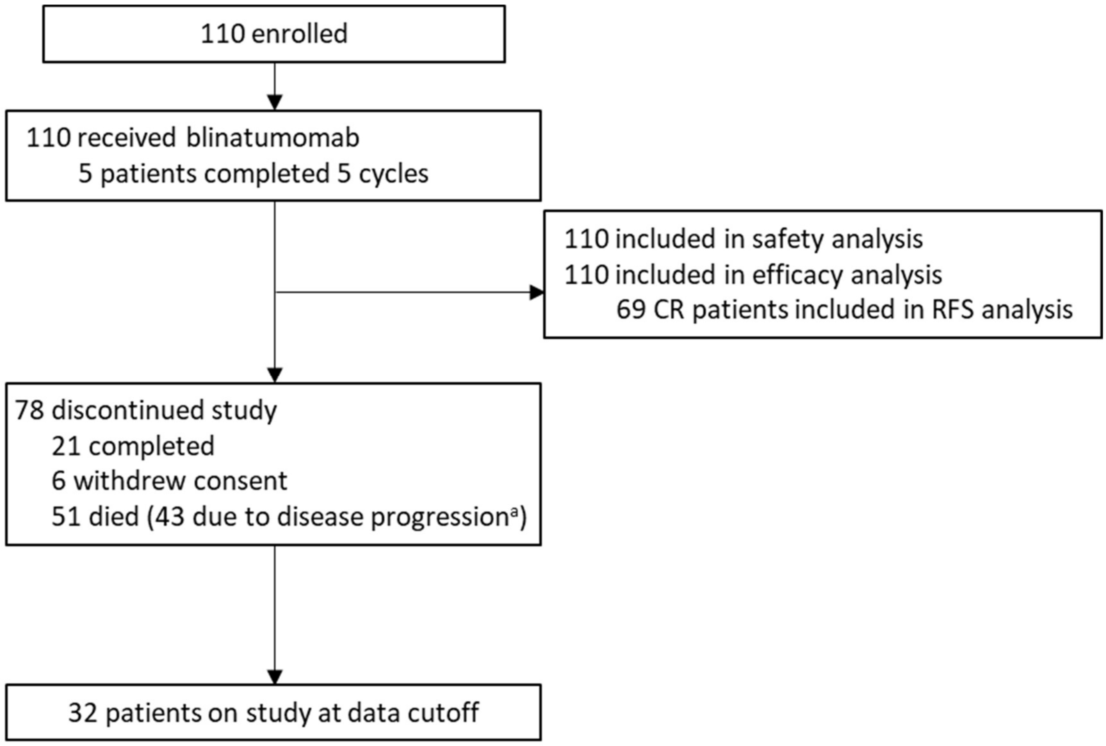

[a]16 of the 51 patients (31.4%) who died by end of study had a clinical response in the first two cycles
RFS, relapse-free survival.

Supplementary Fig. 2. Kaplan-Meier plot of overall survival after HSCT by MRD response status during the first two cycles of blinatumomab (Full Analysis Set - Subjects who had CR)

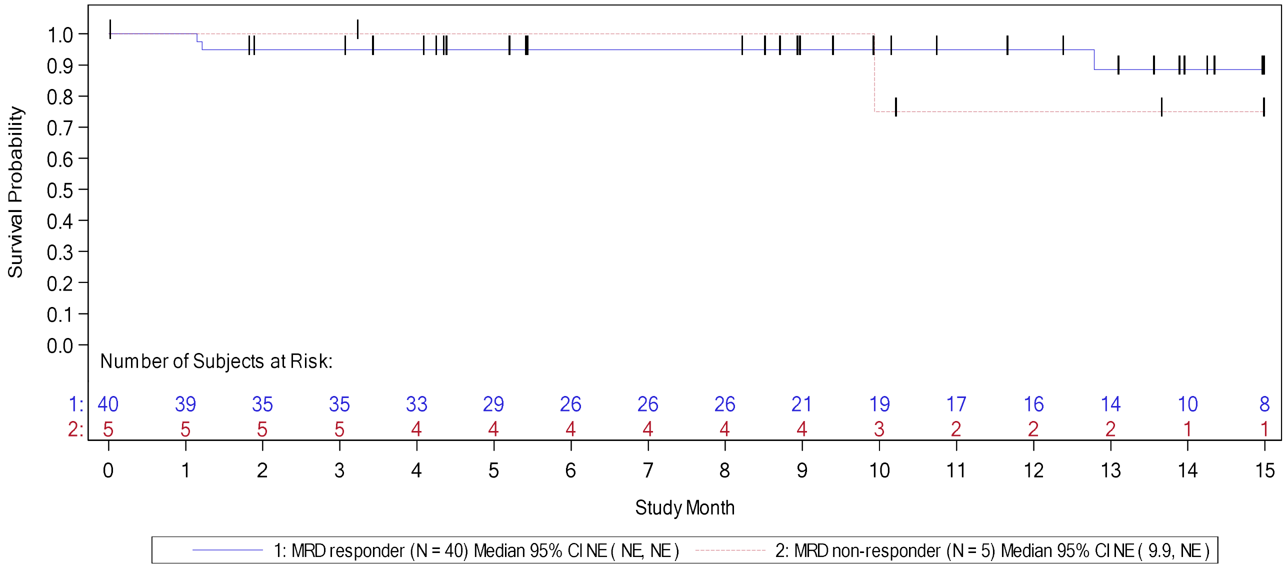

Censor indicated by vertical bar | Months=days/30.5. Figure includes retreatment data (treated as cycle 2) for one subject. Data cutoff date: September 27, 2018.

CI, confidence interval; CR, complete response; HSCT, hematopoietic stem cell transplantation; MRD, minimal residual disease; NE, not estimable.

Supplementary Table 1. Baseline demographics and clinical characteristics.

**Characteristic**	**All patients (N=110)**
Age, median (range), years	8.5 (0.4–17.0)
Age group, years, n (%)	
0–1	13 (12)
2–6	31 (28)
7–17	66 (60)
Sex, n (%)	
Male	62 (56)
Female	48 (44)
Geographic location, n (%)	
Europe	101 (92)
North America	9 (8)
Blast category, n (%)	
<5% with MRD $\geq 10^{-3}$	12 (11)
5–49%	55 (50)
≥50%	42 (38)
Unknown	1 (1)
Current genetic abnormalities[a], n (%)	
MLL rearrangement	18 (16)
t(9;22) BCR-ABL	5 (5)
t(17;19)	2 (2)
t(12;21)/TEL-AML1	9 (8)
Hypo/hyperdiploidy	1 (1)/6 (5)
Other[b]	16 (15)
Constitutional trisomy 21 (Down syndrome), n (%)	4 (4)

Prior therapy, n (%)	
HSCT	45 (41)
Blinatumomab	5 (5)
Disease history, n (%)	
Primary refractory disease	17 (16)
Refractory to reinduction therapy	23 (21)
Second or greater relapse	61 (55)
Relapse after HSCT	44 (40)

[a]More than one type of genetic abnormality can be selected for the same subject. Table includes retreatment data (treated as cycle 2) for one patient.
[b]Includes t(1;19)/E2A-PBX1 (3 patients), IKZF1 deletion (2 patients), t(5;14)/IL3-IGH (1 patient), t(4;11) (Q21;Q23) (1 patient), monosomy 17 (1 patient), N-RAS mutation (1 patient), INPP5D-ABL1 fusion (1 patient), homozygote IKARUS mutation (1 patient), CD10+, CYTDT+, CYIGM, CD34, aberrant co-expression with CD133 (1 patient), and complex karyotype with abnormalities of chromosomes 5, 15, 16, 17 and 19 (1 patient).
HSCT, hematopoietic stem cell transplantation; MLL, mixed lineage leukemia; MRD, minimal residual disease.

Supplementary Table 2. Treatment-emergent and treatment-related adverse events.

**AE, n (%)**	**Treatment emergent**		**Treatment related**	
	**All grades**	**Grade ≥3**	**All grade**	**Grade ≥3**
Any AE	109 (99.1)	71 (64.5)	81 (73.6)	29 (26.4)
**AEs occurring in ≥10% for all grade or ≥5% for grade ≥3**				
Pyrexia	92 (83.6)	15 (13.6)	63 (57.3)	11 (10.0)
Vomiting	30 (27.3)	1 (0.9)	11 (10.0)	0
Headache	27 (24.5)	2 (1.8)	11 (10.0)	2 (1.8)
Cytokine release syndrome	22 (20.0)	2 (1.8)	18 (16.4)	2 (1.8)
Anemia	20 (18.2)	5 (4.5)	3 (2.7)	0
Nausea	20 (18.2)	0	8 (7.3)	0
Cough	19 (17.3)	0	1 (0.9)	0
Pain	18 (16.4)	2 (1.8)	4 (3.6)	1 (0.9)
Hypotension	14 (12.7)	2 (1.8)	5 (4.5)	2 (1.8)
Pain in extremity	14 (12.7)	0	4 (3.6)	0
Abdominal pain	12 (10.9)	0	3 (2.7)	0
Hypokalemia	12 (10.9)	8 (7.3)	4 (3.6)	3 (2.7)
Platelet count decreased	12 (10.9)	11 (10.0)	1 (0.9)	1 (0.9)
Alanine aminotransferase increased	11 (10.0)	7 (6.4)	1 (0.9)	1 (0.9)
Constipation	11 (10.0)	0	1 (0.9)	0
Febrile neutropenia	11 (10.0)	10 (9.1)	3 (2.7)	3 (2.7)
Fluid balance positive	11 (10.0)	0	0	0
Neutropenia	11 (10.0)	8 (7.3)	1 (0.9)	1 (0.9)
Rash	11 (10.0)	0	5 (4.5)	0
Thrombocytopenia	10 (9.1)	5 (4.5)	0	0
**AE categories of interest**				
Infusion reactions	76 (69.1)	12 (10.9)	54 (49.1)	10 (9.1)
Infections	50 (45.5)	20 (18.2)	9 (8.2)	4 (3.6)
Neurologic events	47 (42.7)	6 (5.5)	22 (20.0)	4 (3.6)
Cytopenias	41 (37.3)	32 (29.1)	7 (6.4)	7 (6.4)
Elevated liver enzymes	22 (20.0)	14 (12.7)	4 (3.6)	3 (2.7)
Cytokine release syndrome	22 (20.0)	2 (1.8)	18 (16.4)	2 (1.8)
Decreased immunoglobulins	8 (7.3)	0	5 (4.5)	0
Tumor lysis syndrome	5 (4.5)	3 (2.7)	4 (3.6)	2 (1.8)
Capillary leak syndrome	1 (0.9)	0	0	0

AE, adverse event.

Supplementary Table 3. Best response during the first two cycles of blinatumomab treatment.

**Response**	**Patients with ≥5% blasts at baseline (N=98)**	
	**n**	**% (95% CI)**
CR	58	59.2 (48.8–69.0)
CR with full recovery of peripheral blood counts	39	67.2 (53.7–79.0)
CR with partial recovery of peripheral blood counts	6	10.3 (3.9–21.2)
CR without recovery of peripheral blood counts	13	22.4 (12.5–35.3)
MRD response	46	79.3 (66.6–88.8)
Hypoplastic or acellular bone marrow	1	1.0 (0.0–5.6)
Partial remission	0	0.0 (0.0–3.7)
Stable disease	5	5.1 (1.7–11.5)
Progressive disease	31	31.6 (22.6–41.8)
Not evaluable	1	1.0 (0.0–5.6)
No response data	2	2.0 (0.25–7.2)
	**Patients with <5% blasts at baseline (N=12)**	
MRD response	11	91.7 (61.5–99.8)
No MRD response	0	0
Progressive disease	0	0.0 (0.0–26.5)
No MRD response data	1	8.3 (0.2–38.5)

CR, complete remission; MRD, minimal residual disease.

References

1. Wolfl M, Rasche M, Eyrich M, Schmid R, Reinhardt D, Schlegel PG. Spontaneous reversion of a lineage switch following an initial blinatumomab-induced ALL-to-AML switch in MLL-rearranged infant ALL. Blood Adv. 2018;2(12):1382-5.
